# Multifractal Model for Oromucosal Polymeric Film Performance

**DOI:** 10.3390/pharmaceutics18070875

**Published:** 2026-07-17

**Authors:** Alexandra Barsan (Bujor), Vlad Ghizdovat, Monica Stamate Cretan, Mousa Sha’at, Carmen Anatolia Gafitanu, Ciprian Stamate, Anca Miron, Dragos-Ioan Rusu, Maricel Agop, Lacramioara Ochiuz

**Affiliations:** 1 Department of Pharmaceutical Technology, Faculty of Pharmacy, Grigore T. Popa University of Medicine and Pharmacy, 16 Universității Street, 700115 Iași, Romania; alexandra.m.bujor@umfiasi.ro (A.B.); mousa-shaat@umfiasi.ro (M.S.);; 2 Department of Biophysics and Medical Physics, Faculty of Medicine, Grigore T. Popa University of Medicine and Pharmacy, 16 Universității Street, 700115 Iași, Romania; 3 Department of Mechanical Engineering, Mechatronics and Robotics, Mechanical Faculty, Gheorghe Asachi Technical University of Iasi, Prof. Dr. Docent Dimitrie Mangeron Rd., No. 43, 700050 Iasi, Romania; 4 Department of Pharmacognosy-Phytotherapy, Faculty of Pharmacy, Grigore T. Popa University of Medicine and Pharmacy, 16 Universității Street, 700115 Iași, Romania; 5 Department of Environmental Engineering, Mechanical Engineering and Agritourism, Faculty of Engineering, “Vasile Alecsandri” University of Bacau, 600115 Bacau, Romania; drusu@ub.ro (D.-I.R.);; 6 Romanian Scientists Academy, 54 Splaiul Independentei, 050094 Bucharest, Romania

**Keywords:** oromucosal films, chitosan, hydroxypropyl methylcellulose, swelling kinetics, rupture resistance, elongation behavior, surface roughness, disintegration test, multifractal modeling, drug delivery systems

## Abstract

**Background**: Oromucosal films are thin polymeric dosage forms designed to hydrate rapidly in the oral cavity and enable local or systemic drug delivery. Their performance depends on coupled processes including wetting, swelling, polymer relaxation, matrix softening, and structural failure. Because these phenomena depend strongly on the formulation composition and polymer-network organization, a mechanistic framework linking conventional characterization data to film performance is needed. This study aimed to develop a Madelung-type multifractal swelling–disintegration–release-readiness model for chitosan/hydroxypropyl methylcellulose (HPMC) films and to examine its relevance using a twelve-formulation experimental series. **Methods**: Twelve films based on chitosan (film-forming polysaccharide), HPMC K-4M (hydrophilic swelling polymer), glycerin (plasticizer), and starch (disintegrant) were prepared via solvent casting. The films were characterized for loss on drying, surface pH, mass and thickness uniformity, wetting time, swelling behavior, structural-disintegration onset, elongation response, rupture resistance, folding endurance, and surface roughness. The proposed model described water uptake, swelling-front motion, matrix integrity, local release-readiness activation, and hydration-induced loading as coupled fields across the film thickness. **Results**: Formulation markedly influenced hydration behavior, mechanical performance, structural stability, and surface morphology. Films F2 and F7 emerged as the most promising complementary unloaded matrix platforms for future active-compound incorporation and experimental release evaluation. F2 behaved as a high-swelling, mechanically stable starch-free matrix, whereas F7 combined faster wetting, starch-assisted structural destabilization, and favorable flexibility. **Conclusions**: This framework provides a quantitative link between empirical film characterization and formulation-level mechanistic interpretation. It translates conventional characterization parameters into descriptors related to the apparent water penetration, swelling capacity, matrix-failure tendency, mechanical suitability, and structural heterogeneity. The present results support candidate selection for future Active Pharmaceutical Ingredient-loaded studies but do not constitute validation of drug-release kinetics.

## 1. Introduction

Oromucosal films are solid pharmaceutical forms administered in the oral cavity, where they hydrate, soften, and disperse or dissolve before swallowing or before partial transmucosal absorption, depending on the formulation design and active-compound properties. Their technological advantage is the combination of thin geometry, rapid wetting, dose flexibility, ease of administration, and patient acceptability, particularly when swallowing tablets is difficult [1,2]. A film intended for sublingual or buccal administration must simultaneously satisfy constraints that are not independent: it should be thin and uniform, mechanically strong enough for handling, flexible enough to resist folding, wettable in simulated saliva, capable of swelling without premature fragmentation, and able to disintegrate or erode at a rate consistent with the intended release profile [1,2,3,4].

Polysaccharides and semi-synthetic cellulose derivatives are widely used for mucosal films because they can combine hydration, mucoadhesion, film-forming capacity, and biocompatibility [3]. Chitosan contributes with a cationic character, film formation, and bioadhesive interactions, but increasing chitosan content may also stiffen the network and slow hydration-driven disintegration [5]. HPMC contributes to hydrophilicity, gel formation, and swelling capacity [6], while glycerin acts as a plasticizer by improving chain mobility and reducing brittleness [7]. Starch may act as a disintegrant or hydration-disruption agent, but excessive starch can reduce mechanical integrity and produce friable films [8]. These qualitative mechanisms are well recognized, but formulation development is still frequently driven by empirical screening rather than by a unified model linking the composition, water uptake, swelling, matrix failure, and release [4,9,10,11].

The objective of the present article is to formulate a detailed mathematical framework that can be applied to polymeric films and later extended to active-compound-release kinetics. The model is based on the idea that film structural destabilization and the onset of release readiness are not scalar events but spatiotemporal processes. When a dry film contacts saliva, a hydration front enters the polymer matrix. Water plasticizes the matrix, increases chain mobility, generates swelling pressure, changes local porosity, weakens polymer–polymer contacts, and eventually initiates cracks or pores. This sequence is intrinsically heterogeneous because the film contains thickness variations, surface roughness, loss-on-drying-related volatile/moisture variability, polymer concentration gradients, and disintegrant domains. Therefore, a model that includes only a single first-order disintegration constant cannot represent the full coupled behavior [12]. Conversely, a full molecular simulation is unnecessary for formulation screening. A mesoscopic partial-differential-equation (PDE) model provides an intermediate level of description.

In this context, a Madelung-type multifractal activation–transport structure was adapted to oromucosal films. Here, activation refers to local hydration, structural destabilization, and release readiness within the polymer matrix rather than to measured API release. In simple terms, the Madelung-type contribution makes the model sensitive to the spatial shape of the activation field: smooth, broadly distributed hydration is treated differently from sharply localized hydration fronts or hotspots. In the present application, this curvature-sensitive term is used as a mesoscopic descriptor of heterogeneity and does not imply a quantum-mechanical mechanism in the polymer film [13,14]. The resulting model is not a validated predictive release model; it is applied here to the mechanistic interpretation and formulation ranking of unloaded hydrated matrices.

The present work investigates twelve polysaccharide-based oromucosal film formulations composed of chitosan and HPMC, with and without starch as a disintegrant, to (i) quantify their pharmaco-technical, mechanical, and textural properties, and (ii) propose a Madelung-type multifractal-dynamical framework for the interpretation of swelling, hydration-induced structural destabilization, and mechanical response. In addition, the study aims to identify formulation descriptors that can support future assessments of release-enabling behavior after active-compound incorporation and experimental release testing.

## 2. Materials and Methods

### 2.1. Materials

Chitosan with a medium molecular weight (190,000–310,000 Da) and a degree of deacetylation of 75–85%, potato soluble starch, and rhodamine B (95% purity) were purchased from Sigma Aldrich (Steinheim, Germany). Hydroxypropyl methylcellulose (HPMC K-4M) was obtained from Colorcon (Midland, MI, USA). Glycerin was purchased from ChimReactiv (Bucharest, Romania), while glacial acetic acid was obtained from Chemical Company (Iași, Romania). All chemicals used were of analytical grade.

A simulated isotonic salivary medium was employed for swelling and disintegration experiments. The salivary medium was prepared by dissolving disodium phosphate, dipotassium phosphate, and sodium chloride, all purchased from Sigma Aldrich (Steinheim, Germany), in distilled water, followed by pH adjustment to 6.8 with 85% phosphoric acid (Sigma Aldrich, Steinheim, Germany).

### 2.2. Experimental Design and Film Formulation

Twelve oromucosal films were prepared according to a three-variable quantitative formulation plan. Chitosan was used as a film-forming polysaccharide at 0.5% or 1.0%. HPMC K-4M was used as a second film-forming polymer at 1.0% or 1.5%. Starch was included as a disintegrant at 0%, 0.05%, or 0.10%. Glycerin was fixed at 1.0% in all formulations as a plasticizer. The design allowed comparisons of four polymer bases without starch and eight corresponding starch-containing films. The film codes used throughout the manuscript are shown in Table 1, and concentrations are given as percentage values used in the casting solutions. At this stage, the films did not contain any API.

### 2.3. Preparation of Oromucosal Films

Films were obtained using the solvent casting method. Chitosan was dissolved in 1% *v*/*v* acetic acid under magnetic stirring and centrifuged to remove the undissolved fraction. HPMC was gradually added under continuous stirring. The resulting macromolecular solution was then sonicated to remove entrapped air bubbles. Starch (when present) was added in the final mixing step, followed by glycerin incorporation to improve flexibility and prevent brittleness. The resulting macromolecular solutions were cast into Petri dishes and dried at 35 °C for 6 h until complete solvent evaporation. Dried films were carefully peeled off and cut into standardized specimens. Films were stored in a desiccator to minimize humidity-driven variability prior to analysis [15,16].

### 2.4. Pharmacotechnical Characterization

The pharmacotechnical characterization comprised loss on drying, surface pH, mean film mass, mean film thickness, in vitro wetting time, in vitro structural-disintegration onset time, and swelling kinetics. The starch-free formulations F1–F4 were considered reference matrices for assessing the effect of starch.

#### 2.4.1. Loss on Drying

Loss on drying was determined according to Ph. Eur., 12th edition [17]. Briefly, the weighing bottle was previously dried to a constant mass under the same experimental conditions as the sample. Film samples were placed in the weighing bottle and dried in an oven (DIN 12880, Binder, Tuttlingen, Germany) at 105 °C until a constant mass was reached. The constant mass was defined as a mass difference of not more than 0.5 mg between two consecutive weighings performed at 30 min intervals. Before weighing, samples were cooled in a desiccator.

Loss on drying was expressed as the percentage mass loss. If $M_{0,i}$ is the initial film mass and $M_{d,i}$ is the dried mass of formulation i, the loss on drying is as follows:

(1) $${LOD}_{i}\left(\%\right)=\frac{M_{0,i}-M_{d,i}}{M_{0,i}}\times 100$$

Loss on drying is a gravimetric mass-loss endpoint and is not water-specific. Water is expected to contribute substantially in these hydrophilic films, but other removable volatile components may also contribute under the specified drying conditions. Accordingly, the parameter is interpreted as loss on drying rather than as a direct measurement of the water content. The volatile/moisture-associated fraction may nevertheless influence the handling, swelling onset, and apparent mechanical behavior through matrix plasticization and changes in intermolecular interactions.

#### 2.4.2. Surface pH

The surface pH of the films was determined after allowing the samples to swell in 5 mL of distilled water for 30 min [18]. The pH was then measured using a portable pH meter equipped with a probe (Testo 206, Testo, Sparta, NJ, USA). Measurements were performed in triplicate, and the results were expressed as the mean ± SD. For oromucosal formulations, near-neutral pH is desirable to minimize mucosal irritation and avoid discomfort.

#### 2.4.3. Mass and Thickness Uniformity

The thickness and mass uniformity of precisely cut 1 cm × 1 cm film samples, taken from each formulation, were evaluated using a digital micrometer (DIN863, Messzeuge GmbH, Spangenberg, Germany) and an analytical balance (XS 105 Dual Range, Mettler Toledo, Greifensee, Switzerland), respectively. For each formulation, five film samples were weighed (mg), and the mean mass and SD were calculated. Film thickness was measured at five different points (µm), and the results were expressed as the mean thickness and SD [19]. The relative standard deviation ${RSD}_{i}$ of a generic descriptor $X_{i}$ was calculated as follows:

(2) $${RSD}_{i}\left(\%\right)=\frac{s_{i}}{{\bar{X}}_{i}}\times 100$$

where ${\bar{X}}_{i}$ is the mean and $s_{i}$ is the standard deviation.

#### 2.4.4. In Vitro Wetting Time

Wetting time was determined using rhodamine B solution, 0.05%, as a water-soluble dye marker. A piece of absorbent paper placed in a Petri dish was impregnated with 6 mL of 0.05% *w*/*v* aqueous rhodamine B solution. A 1 cm × 1 cm film sample was then placed on the wetted paper, and the time required for the dye to diffuse through the film and appear on its upper surface was recorded in seconds [20]. Measurements were performed in triplicate, and the results were expressed as the mean ± SD. The wetting time $t_{w,i}$ was recorded as the time required for visible hydration of formulation i in simulated salivary medium. Wetting is an early event that controls the onset of swelling, matrix plasticization, and eventual disintegration. A shorter $t_{w,i}$ indicates faster saliva penetration and faster activation of the hydrated polymer network.

#### 2.4.5. In Vitro Structural-Disintegration Onset Time

The structural-disintegration behavior of the films was evaluated under simulated physiological conditions. Briefly, each film sample was placed in an Erlenmeyer flask containing 25 mL of simulated salivary medium (pH 6.8) maintained at 37 °C under magnetic stirring. The assessment criterion was defined as the time at which the first clearly visible and persistent macroscopic fissure or localized loss of structural integrity appeared on the film surface. Simulated salivary medium was prepared by dissolving disodium phosphate (0.238%), dipotassium phosphate (0.019%), and sodium chloride (0.8%) in distilled water and adjusting the pH to 6.8 with phosphoric acid, as previously reported by Koland et al. [21]. Measurements were performed in triplicate, and the results were expressed in seconds (s) as the mean ± SD. The structural-disintegration onset time $t_{d,i}$ was recorded as the time at which the first visible crack appeared on the film surface. This definition is important because it captures the onset of mechanical matrix failure rather than complete dissolution. In this context, $t_{d,i}$ is interpreted as an experimental proxy for the inverse of the local onset disintegration-rate constant:

(3) $$k_{d,i}^{proxy}=\frac{1}{t_{d,i}}$$

This operational endpoint is termed structural-disintegration onset time throughout the manuscript and should not be interpreted as complete film dissolution or complete disappearance of the dosage form.

#### 2.4.6. Swelling Kinetics and Maximum Swelling

Swelling was evaluated by immersing pre-weighed film samples in simulated isotonic salivary medium and monitoring the mass increase at predetermined time intervals. Briefly, the films were immersed in 30 mL of medium at 37 °C, removed at the selected time points, gently blotted with absorbent paper to eliminate excess surface liquid, and reweighed [21,22].

The swelling percentage was evaluated after immersion in simulated salivary medium. Measurements were performed in triplicate, and the swelling kinetics were plotted as a function of time.

If $M_{t,i}$ is the hydrated mass at time t and $M_{0,i}$ is the initial dry mass, the swelling percentage is as follows:

(4) $$S_{i}\left(t\right)\left(\%\right)=\frac{M_{t,i}-M_{0,i}}{M_{0,i}}\times 100$$

The maximum swelling percentage is as follows:

(5) $$S_{max,i}=\underset{t\in \left[0,T\right]}{max}S_{i}\left(t\right)$$

### 2.5. Mechanical Testing

Mechanical testing was performed to assess elongation behavior, post-elongation thickness reduction, rupture resistance, and folding endurance. These endpoints are essential for film handling, packaging, cutting, administration, and patient use.

The mechanical behavior of the films was evaluated via puncture-based elongation and rupture testing using a tribometer equipped with a pin-on-disc system [23] (CETR UMT-2, Schaefer Campbell, Campbell, CA, USA; force range 10 µN–20 N). Film thickness was measured with a digital micrometer (DIN863, Messzeuge GmbH, Spangenberg, Germany; measuring range 0–25 mm, precision 0.001 mm).

All tests were performed under controlled ambient conditions at 29 °C and 38.5% relative humidity. Film samples were fixed via adhesion onto a circular holder with an outer diameter of 24 mm and an inner opening of 10 mm, corresponding to an exposed test area of 78.5 mm^2^. Elongation behavior was assessed using a spherical pin with a diameter of 6.36 mm, whereas rupture testing was performed using a flat-tipped conical pin with a diameter of 1.58 mm.

#### 2.5.1. Elongation Force and Post-Elongation Thickness Reduction

The elongation force $F_{e,i}$ represents the force required to stretch the film under the specified test conditions.

The post-elongation thickness reduction $R_{h,i}$ was calculated as follows:

(6) $$R_{h,i}\left(\%\right)=\frac{h_{0,i}-h_{e,i}}{h_{0,i}}\times 100$$

where $h_{0,i}$ is the initial thickness and $h_{e,i}$ is the film thickness after elongation. High $R_{h,i}$ indicates pronounced plastic deformation or chain mobility during stretching.

#### 2.5.2. Rupture Resistance

Rupture resistance was expressed as force per cross-sectional area:

(7) $$\tau_{r,i}=\frac{F_{r,i}}{A_{i}},$$

where $F_{r,i}$ is the rupture force and $A_{i}$ is the contact area of the flat-tipped conical pin tip.

#### 2.5.3. Folding Endurance

Folding endurance was determined by repeatedly folding film samples (1 cm × 3 cm) at the same point to an angle of 180° until rupture or up to a maximum of 300 folds. The number of folds causing visible cracking was recorded as the folding endurance [21]. Folding endurance contributes to proper adaptation and contact with the mucosal surface. In addition, it represents an important handling-related component of the model, since a film that hydrates and disintegrates rapidly but lacks sufficient mechanical integrity during manipulation would not be suitable as a pharmaceutical dosage form.

### 2.6. Surface Roughness Analysis

The surface roughness of the films was evaluated using a Form Talysurf Intra 50 surface profilometer (Taylor Hobson, Leicester, UK) equipped with a 112/2009 standard conical diamond stylus (tip radius 2 µm, tip angle 90°). Film surface scans were performed over a length of 5 cm to assess surface texture and micron-scale irregularities [24,25,26]. Measurements were performed in duplicate. Roughness profiles were recorded, and the average roughness was calculated by integrating the absolute values of the peaks and valleys over the sampling length and then dividing this sum by the sampling length [27].

The formula for the average roughness (Ra) is given by the following equation:

$$Ra=\frac{1}{l}\int_{0}^{l}\left|Z(x)\right|dx$$

where *l* represents the sampling length, *x* represents the distance from the measurement point to the origin, and *Z*(*x*) represents the deviation of the surface from the mean line.

Roughness is mechanistically relevant because it changes the effective contact area with saliva, promotes local water entry, and can generate spatial heterogeneity in swelling and disintegration fronts. The surface-texture information summarized in Appendix A Table A2 indicates that starch generally increased average roughness.

### 2.7. Mathematical Model Construction

Input variables were selected according to three predefined criteria: (i) availability of the descriptor for all twelve formulations, (ii) direct mechanistic relevance to water penetration, swelling, matrix deformation or failure, or structural heterogeneity, and (iii) sufficient interpretability for formulation-level comparison. Variables were excluded from quantitative model-derived indices when complete formulation-specific numerical data were unavailable or when their relationship with the present unloaded-film system could not be parameterized from the available experiments.

#### 2.7.1. Spatial Domain and State Variables

Each film was represented as a hydrated polymer slab of thickness $h_{i}$. The spatial coordinate x was defined across the film thickness:

(8) $$x\in \left[0,h_{i}\right],\;\;t\geq 0$$

For each formulation i∈{F1,…,F12}, the model defines five fields:

$$w_{i}\left(x,t\right)-\mathrm{water}\;\mathrm{or}\;\mathrm{saliva}\;\mathrm{content},$$

$$\rho_{i}\left(x,t\right)-\mathrm{release}/\mathrm{disintegration}\;\mathrm{activation}\;\mathrm{density},$$

$$u_{i}\left(x,t\right)-\mathrm{effective}\;\mathrm{swelling}\text{-}\mathrm{front}\;\mathrm{or}\;\mathrm{activation}\text{-}\mathrm{front}\;\mathrm{velocity},$$

$$\sigma_{i}\left(x,t\right)-\mathrm{hydration},\;\mathrm{swelling}\;\mathrm{or}\;\mathrm{matrix}\text{-}\mathrm{loading}\;\mathrm{field},$$

$$m_{i}\left(x,t\right)\in \left[0,1\right]-\mathrm{matrix}\text{-}\mathrm{integrity}\;\mathrm{field}.$$

The limiting values of $m_{i}\left(x,t\right)$ have direct physical meaning:

$$m_{i}\left(x,t\right)=1\;\mathrm{intact}\;\mathrm{matrix},\;\;m_{i}\left(x,t\right)=0\;\mathrm{fully}\;\mathrm{disintegrated}\;\mathrm{matrix}$$

#### 2.7.2. Water Penetration Equation

Water penetration was modeled using a nonlinear diffusion equation:

(9) $$\frac{\partial w_{i}}{\partial t}=\frac{\partial }{\partial x}\left[D_{w,i}\left(m_{i},\rho_{i}\right)\frac{\partial w_{i}}{\partial x}\right]-k_{b,i}w_{i}.$$

Here, $D_{w,i}$ is the effective water diffusivity, which can depend on the local matrix integrity and activation state, and $k_{b,i}$ is an apparent water-binding or immobilization coefficient. A simple experimental proxy for $D_{w,i}$ can be constructed from the film thickness and wetting time:

(10) $$D_{w,i}^{proxy}=\frac{h_{i}^{2}}{t_{w,i}}$$

This proxy is not a true diffusivity because wetting time is a macroscopic observational endpoint, but it is useful for formulation ranking.

#### 2.7.3. Activation-Density Balance

The activation-density field $\rho_{i}\left(x,t\right)$ describes local readiness for disintegration and active-compound release. It increases when hydration and swelling exceed critical thresholds and decreases when the matrix erodes or fully disintegrates:

(11) $$\frac{\partial \rho_{i}}{\partial t}+\frac{\partial }{\partial x}\left(\rho_{i}u_{i}\right)=S_{i}\left(w_{i},\sigma_{i},m_{i}\right)-\lambda_{i}\rho_{i}-E_{i}\left(\rho_{i},w_{i},m_{i}\right)$$

The source term $S_{i}$ can be written as a cooperative threshold function:

(12) $$S_{i}\left(w_{i},\sigma_{i},m_{i}\right)=A_{i}\;H\left(w_{i}-w_{c,i}\right)\;H\left(\sigma_{i}-\sigma_{c,i}\right)\;m_{i}$$

A smooth logistic alternative is as follows:

(13) $$S_{i}\left(w_{i},\sigma_{i},m_{i}\right)=A_{i}{\left[1+exp\{-a_{w,i}\left(w_{i}-w_{c,i}\right)\}\right]}^{-1}{\left[1+exp\{-a_{\sigma ,i}\left(\sigma_{i}-\sigma_{c,i}\right)\}\right]}^{-1}m_{i}$$

#### 2.7.4. Swelling-Front Velocity Equation

The velocity field $u_{i}\left(x,t\right)$ represents the propagation of the hydration/disintegration front. Its evolution is governed by gradients in a formulation-dependent effective potential:

(14) $$\frac{\partial u_{i}}{\partial t}+u_{i}\frac{\partial u_{i}}{\partial x}=-\frac{\partial }{\partial x}\left[V_{i}\left(x\right)+Q_{i}\left(\rho_{i}\right)+\Phi_{i}\left(w_{i},\sigma_{i}\right)\right]-\gamma_{i}u_{i}$$

Here, $\gamma_{i}$ is a damping coefficient representing viscoelastic relaxation, $V_{i}\left(x\right)$ is the structural heterogeneity potential, $Q_{i}\left(\rho_{i}\right)$ is the Madelung-type curvature term, and $\Phi_{i}\left(w_{i},\sigma_{i}\right)$ is the hydration-loading contribution.

#### 2.7.5. Madelung-Type Curvature Term

The Madelung-type contribution was defined as follows:

(15) $$Q_{i}\left(\rho_{i}\right)=-D_{f,i}^{2}\frac{\partial_{xx}\sqrt{\rho_{i}+\varepsilon }}{\sqrt{\rho_{i}+\varepsilon }}$$

The small parameter ε>0 prevents singular behavior at a low activation density. The coefficient $D_{f,i}$ is interpreted as a multifractal or heterogeneity-sensitive transport coefficient. In these films, larger $D_{f,i}$ values correspond to stronger spatial localization of swelling, pore opening, or crack initiation. Starch-containing films and rougher films are expected to have higher effective heterogeneity and therefore potentially larger $D_{f,i}$.

#### 2.7.6. Loading or Swelling-Stress Field

The loading field $\sigma_{i}\left(x,t\right)$ represents hydration pressure, osmotic swelling stress, polymer relaxation stress, and local mechanical destabilization:

(16) $$\frac{\partial \sigma_{i}}{\partial t}=L_{i}w_{i}+\int_{0}^{h_{i}}K_{i}\left(x-x^{\prime }\right)\rho_{i}\left(x^{\prime },t\right)\;dx^{\prime }-\eta_{i}\rho_{i}-\omega_{i}\sigma_{i}$$

The term $L_{i}w_{i}$ represents hydration-driven loading. The kernel $K_{i}$ represents nonlocal coupling through the hydrated polymer network. The term $\eta_{i}\rho_{i}$ represents stress release caused by local disintegration, and $\omega_{i}\sigma_{i}$ represents relaxation.

#### 2.7.7. Matrix-Integrity Equation

Matrix integrity was modeled by the following:

(17) $$\frac{\partial m_{i}}{\partial t}=-k_{m,i}H\left(w_{i}-w_{m,i}\right)H\left(\sigma_{i}-\sigma_{m,i}\right)m_{i}$$

A smooth version is as follows:

(18) $$\frac{\partial m_{i}}{\partial t}=-k_{m,i}{\left[1+exp\{-b_{w,i}\left(w_{i}-w_{m,i}\right)\}\right]}^{-1}{\left[1+exp\{-b_{\sigma ,i}\left(\sigma_{i}-\sigma_{m,i}\right)\}\right]}^{-1}m_{i}$$

This equation captures irreversible matrix weakening after sufficient hydration and swelling stress.

#### 2.7.8. Structural Heterogeneity Potential

The formulation-dependent potential $V_{i}\left(x\right)$ was decomposed as follows:

(19) $$V_{i}\left(x\right)=V_{C,i}\left(x\right)+V_{H,i}\left(x\right)+V_{A,i}\left(x\right)+V_{G,i}\left(x\right)+V_{R,i}\left(x\right)+V_{h,i}\left(x\right)$$

The components denote contributions from chitosan C, HPMC H, starch A, glycerin G, roughness R, and thickness h. For formulation-level ranking, a reduced scalar approximation is as follows:

(20) $${\bar{V}}_{i}=\alpha_{C}C_{i}+\alpha_{H}H_{i}+\alpha_{A}A_{i}+\alpha_{G}G_{i}+\alpha_{R}R_{i}+\alpha_{h}h_{i}+\alpha_{L}{LOD}_{i}$$

The raw experimental inputs corresponding to $C_{i}$, $H_{i}$, $A_{i}$, and $G_{i}$ are formulation percentages, $R_{i}$ is the average roughness, $h_{i}$ is thickness, and ${LOD}_{i}$ is loss on drying. The coefficients $\alpha_{j}$ must be estimated experimentally after active-compound release data become available; for Equation (20), the raw descriptors are converted to normalized dimensionless scores as specified below.

In the reduced scalar approximation of Equation (20), the loss-on-drying contribution is retained as a formulation-level descriptor only; no spatially resolved loss-on-drying field is assumed in Equation (19). To avoid the direct combination of variables with different native units, each scalar input to Equation (20) is first min–max normalized within the twelve-formulation dataset. The corresponding coefficients therefore act on dimensionless scores.

Importantly, the mean roughness parameter Ra is not identified directly with the spatial heterogeneity potential $V_{i}\left(x\right)$. In the present dataset, mean roughness is used only as a formulation-level proxy for the magnitude of surface-related heterogeneity.

#### 2.7.9. Release Flux for Future API-Loaded Films

For future active-loaded films, the mobile active concentration $c_{i}\left(x,t\right)$ can be coupled to a matrix state:

(21) $$\frac{\partial c_{i}}{\partial t}=\frac{\partial }{\partial x}\left[D_{c,i}\left(w_{i},m_{i}\right)\frac{\partial c_{i}}{\partial x}\right]-k_{bind,i}c_{i}+k_{rel,i}\rho_{i}$$

The release flux at the film surface is as follows:

(22) $$J_{i}\left(t\right)=-D_{c,i}\left(w_{i},m_{i}\right){\frac{\partial c_{i}}{\partial x}|}_{x=0,h_{i}}$$

The cumulative released fraction is as follows:

(23) $$F_{i}\left(t\right)=\frac{1}{M_{\infty ,i}}\int_{0}^{t}\left[J_{i}\left(0,\tau \right)+J_{i}\left(h_{i},\tau \right)\right]A_{s}\;d\tau$$

Here, $A_{s}$ is the exposed surface area and $M_{\infty ,i}$ is the total releasable amount. Equations (21)–(23) are not calibrated in the present work because the films are unloaded.

#### 2.7.10. Hydration–Disintegration Activity Index

To provide a transparent formulation-level ranking from the available experimental data, the hydration–disintegration activity index was defined as follows:

(24) $${HDAI}_{i}=0.35\;{\hat{S}}_{max,i}+0.25(1-{\hat{\tau }}_{w,i})+0.25\;(1-{\hat{\tau }}_{d,i})+0.15\;{\hat{M}}_{i}$$

The mechanical suitability term was calculated as follows:

(25) $${\hat{M}}_{i}=0.60\;{\hat{\tau }}_{r,i}+0.40\;{\hat{F}}_{fold,i}.$$

All hatted quantities are min–max normalized descriptors. Maximum swelling and mechanical suitability were treated as benefit-type criteria, for which higher values are favorable. The wetting time and structural-disintegration onset time were treated as cost-type criteria, for which lower values are favorable; therefore, their contributions were expressed as complementary normalized scores, $1-{\hat{t}}_{w,i}$ and $1-{\hat{t}}_{d,i}$, respectively. For a generic descriptor $X_{i}$, min–max normalization was performed as ${\hat{X}}_{i}=\left(X_{i}-X_{min}\right)/\left(X_{max}-X_{min}\right)$. The HDAI is not a regulatory endpoint or a validated release metric. It is a model-readiness descriptor that combines the swelling capacity, rapid wetting, early structural-disintegration onset, and mechanical suitability.

For reproducibility, folding-endurance results reported as >300 cycles were conservatively encoded as 300 cycles before min–max normalization; equivalently, $F_{fold,i}\left(300\right)=min\left(F_{fold,i},300\right)$, and the normalized folding term in Equation (25) is calculated from this capped variable. The weighting vector (0.35, 0.25, 0.25, 0.15) was specified a priori as a heuristic screening choice and was not optimized against API-release data. Maximum swelling received the largest weight because hydration-driven network expansion is central to the present unloaded-film dataset; wetting and structural-disintegration onset received equal intermediate weights, while mechanical suitability was retained as a lower-weight handling constraint.

### 2.8. Statistical Analysis

The experimental values are reported as the mean ± standard deviation, as reported in Tables 2–4. Statistical analysis was performed among formulations using one-way analysis of variance (ANOVA), followed by Tukey’s post hoc multiple-comparison test using GraphPad 5.0. Differences were considered statistically significant at *p* < 0.05. No inferential model was fitted to active-compound release because no active release data were available. The model-derived quantities $D_{w,i}^{proxy}$, $k_{d,i}^{proxy}$, and ${HDAI}_{i}$ were calculated deterministically from the reported experimental means.

Tukey-adjusted significance outcomes are indicated in the tables by an asterisk when adjusted *p* < 0.05. The model-derived proxies and HDAI were calculated from experimental means without the propagation of replicate-level uncertainty; the replicate number does not alter the mathematical structure of the PDE framework but does affect the precision of derived quantities and ranking stability.

## 3. Results

### 3.1. Formulation Feasibility and General Film Properties

All twelve formulations yielded solvent-cast films suitable for pharmacotechnical and mechanical characterization. The formulation design confirms the central technological role of viscosity and mechanical integrity. Polymer percentages had to remain compatible with casting before gelation. Starch was restricted to 0.05% and 0.10% because higher percentages produced friable films. Glycerin at 1.0% was sufficient to produce flexible films detachable from Petri plates after drying.

### 3.2. Loss on Drying, Surface pH, Mass, and Thickness

The loss-on-drying, surface pH, mass, and thickness results are summarized in Table 2.

**Table 2:** Loss on drying, surface pH, mass, and thickness of the films.

Film	Loss on Drying (%)	Surface pH	Mass for 1 cm^2^ (mg)	Thickness (µm)
F1	22.3 ± 0.9	6.15 ± 0.05	20.7 ± 1.4	53.1 ± 4.5
F2	16.2 ± 0.8	6.22 ± 0.06	22.7 ± 1.5	57.2 ± 2.9
F3	18.5 ± 0.5	5.91 ± 0.04	21.2 ± 1.6	70.5 ± 5.1
F4	15.7 ± 0.7	6.33 ± 0.09	25.1 ± 1.4	73.5 ± 4.5
F5	24.2 ± 0.6 *	6.29 ± 0.07	23.7 ± 1.6	54.5 ± 5.5
F6	23.5 ± 0.5	6.48 ± 0.04 *	24.8 ± 1.8 *	78.5 ± 2.5 *
F7	17.4 ± 0.3	6.44 ± 0.05 *	25.8 ± 1.6	80.5 ± 4.2 *
F8	18.4 ± 0.5 *	6.56 ± 0.04 *	26.4 ± 1.6 *	92.5 ± 4.5 *
F9	20.1 ± 0.7	6.35 ± 0.03 *	27.5 ± 1.7 *	83.2 ± 3.9 *
F10	21.6 ± 0.8 *	6.44 ± 0.07 *	28.6 ± 1.6 *	91.1 ± 4.5 *
F11	19.7 ± 0.3 *	6.41 ± 0.05	30.8 ± 1.2 *	85.5 ± 5.5
F12	20.2 ± 0.4 *	6.52 ± 0.09 *	31.6 ± 1.2 *	93.5 ± 4.8 *

Values are expressed as the mean ± SD. Starch-containing formulations were compared with their corresponding starch-free reference: F5–F6 vs. F1, F7–F8 vs. F2, F9–F10 vs. F3, and F11–F12 vs. F4. * *p* < 0.05.

The pharmacotechnical properties varied as a function of the formulation composition. Film mass and thickness increased overall with the polymer and starch content, ranging from 20.7 to 31.6 mg and from 53.1 to 93.5 µm, respectively.

Surface pH remained within a narrow interval (5.91–6.56), indicating acceptable compatibility with the oral mucosa. Minor differences were observed between formulations, indicating that changes in the polymer composition had a limited effect on the final surface pH of the films.

Loss on drying ranged from 15.7% to 24.2%, with higher values observed in some starch-containing formulations, particularly F5 and F6. Because the method is gravimetric and not water-specific, these values should be interpreted as removable volatile mass under the specified drying conditions rather than as direct moisture content. Water is expected to be a major contributor in these hydrophilic matrices, but contributions from other residual volatile components cannot be excluded. The higher values in F5 and F6 may reflect a stronger association of a volatile/moisture fraction with the hydrophilic starch-containing matrix and a less dense polymer network; any relationship with their lower puncture resistance or folding endurance remains a mechanistic hypothesis rather than a demonstrated causal effect.

The volatile/moisture-associated fraction can influence film performance because water can plasticize polymer networks and alter brittleness, tackiness, mechanical behavior, swelling, and adhesion. However, the selective quantification of water would require a water-specific analytical method, such as Karl Fischer titration. Accordingly, the present loss-on-drying values are used as comparative formulation descriptors and not as direct measurements of the water content.

### 3.3. Wetting Time, Swelling, and Structural-Disintegration Onset

The wetting time, maximum swelling, and structural-disintegration onset time are summarized in Table 3.

Wetting indicates the initial hydration step of the film. Lower wetting times reflect faster liquid penetration and earlier matrix activation. A shorter wetting time indicates faster saliva penetration and faster activation of the hydrated polymer network. The wetting time varied considerably among the formulations, ranging from 7.3 ± 1.2 s for F5 to 26.6 ± 1.5 s for F4 (Table 3), indicating a strong influence of the formulation composition on the initial hydration behavior of the films. The starch-free formulations containing higher polymer content exhibited slower wetting, consistent with the formation of a denser matrix that delayed liquid penetration. The addition of starch generally reduced the wetting time, although the magnitude of this effect depended on the initial polymer composition. This effect was most evident in the formulations containing 0.5% chitosan, where F5, F6, F7, and F8 all showed lower wetting times than their starch-free counterparts F1 and F2. These findings indicate that starch facilitated the initial uptake and diffusion of water through the films. This effect may be attributed not only to the formation of a less compact and more heterogeneous matrix, but also to the intrinsic hydrophilicity of starch, which can promote rapid water absorption at the film surface. By contrast, higher concentrations of chitosan and HPMC appeared to delay wetting, probably due to the formation of a more cohesive polymer network that limited rapid liquid penetration.

The recorded disintegration-related parameter reflected the time to the first visible fissure on the film surface rather than complete film disintegration indicating the onset of structural destabilization rather than total film disintegration. The parameter varied markedly among formulations, ranging from 178.6 ± 3.3 s for F7 to 2160.3 ± 23.3 s for F4, showing a strong influence of the formulation composition on film integrity in simulated salivary medium. In general, formulations containing 0.5% chitosan showed much shorter times to structural failure than those containing 1.0% chitosan, suggesting that the higher chitosan content offered greater resistance. Among the starch-free films, the onset of structural disintegration was substantially delayed as the polymer content increased. The effect of starch was composition-dependent but, overall, starch tended to accelerate the onset of structural disintegration. In the formulations containing 1.0% chitosan, starch caused a pronounced reduction in structural stability: F9–F12 exhibited markedly shorter times to fissure formation than F3 and F4.

Although both polymers are hydrophilic, increasing the polymer concentration may prolong the onset of structural disintegration due to the formation of a viscous gel layer after wetting, as previously reported by Panraksa et al. [28].

Starch addition generally reduced the time to structural disintegration, with a more pronounced effect at 0.05% than at 0.1%. This behavior may be related to the hydrophilic nature of starch, whose hydroxyl groups promote water uptake through hydrogen-bonding interactions, as also reflected by the shorter wetting times observed for films containing 0.05% starch. The hydrophilic properties of starch are largely influenced by its amylose content [29]. Since starch hydrolysis may additionally contribute to faster film destabilization under physiological conditions, its functional role may be even more relevant in vivo in the presence of salivary amylase [30].

**Figure 1:**
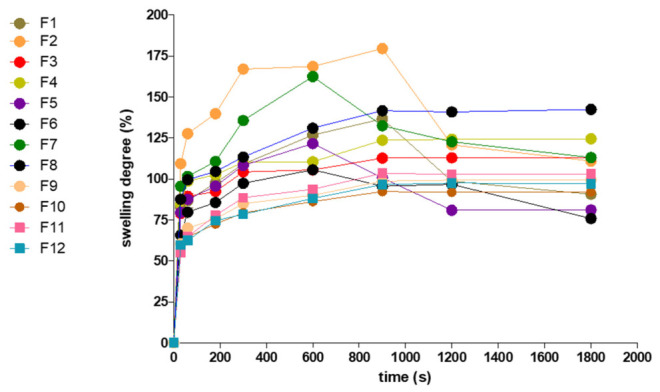
Swelling kinetics of polysaccharide-based oromucosal films (F1–F12) in simulated salivary medium.

The swelling kinetics of the films are shown in Figure 1. All formulations exhibited rapid initial hydration, with a marked increase in the swelling degree during the first minute of immersion. The maximum swelling degrees (Table 3) ranged from 91.1% (F10) to 177.8% (F2) and were obtained after 10 min. Among the starch-free films, F2 showed the highest swelling. In general, films containing 0.5% chitosan showed greater swelling than those containing 1.0% chitosan, particularly when combined with 1.5% HPMC, suggesting that a lower chitosan content favored water penetration and matrix expansion. This is consistent with previous reports indicating that the limited water solubility of chitosan may restrict film swelling, as observed in ondansetron-loaded chitosan/PVP K30 films [21].

Starch addition affected swelling in a formulation-dependent manner: in some systems it reduced maximum swelling, while in others, particularly F7 and F8, high swelling values were maintained. Formulations F1, F2, F5, F6, and F8 exhibited a decline in the swelling percentage over time, consistent with progressive matrix breakdown.

Table 3 and Figure 1 show that F3, F4, F7, F9, F10, F11, and F12 reached a swelling-equilibrium state within approximately 15 min. In contrast, F1, F2, F5, F6, and F8 displayed a subsequent decrease in the swelling percentage, attributed to advanced disintegration and polymeric mass loss. Mechanistically, these two behaviors correspond to different regimes:

(26) $$the\;\mathrm{equilibrium}\text{-}\mathrm{swelling}\;\mathrm{regime}\;\frac{dS_{i}\left(t\right)}{dt}\to 0\;$$

and

(27) $$\mathrm{the}\;\mathrm{swelling}\text{-}\mathrm{loss}/\mathrm{disintegration}\;\mathrm{regime}\;\frac{dS_{i}\left(t\right)}{dt}<0\;$$

The highest swelling in the first 60 s was reported for F2, while the lowest early swelling was reported for F12. This supports the interpretation that F2 is a rapidly hydrating and strongly swelling matrix, whereas F12 is a thicker, mechanically persistent and slower-disintegrating matrix.

The effect of starch was formulation-dependent. Starch-containing films generally wetted faster than their corresponding starch-free films, consistent with the role of starch as a disintegration-promoting excipient. However, starch also reduced maximum swelling relative to the corresponding starch-free films. This suggests that starch promoted earlier structural disruption and water ingress but reduced the ability of the hydrated network to maintain a high-swelling gel state.

Among starch-free formulations, the order of structural-disintegration onset time was as follows:

F2<F1<F3<F4

Among starch-containing formulations, the order was as follows:

F7<F5<F8<F6<F9<F11<F10<F12

**Figure 2:**
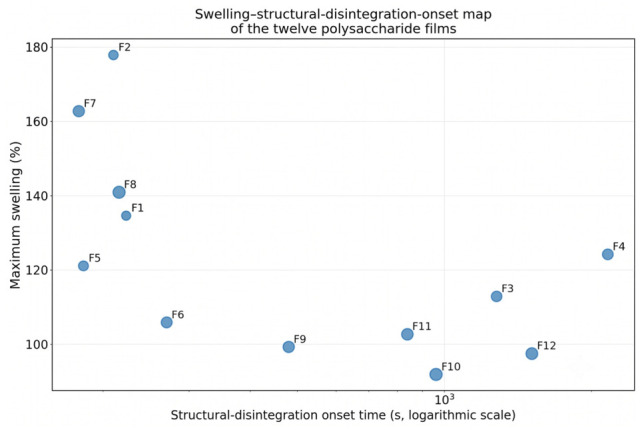
Swelling–structural-disintegration-onset map of the twelve films. Bubble size is proportional to film thickness. F2 and F7 occupy the region combining high swelling with relatively short structural-disintegration onset time.

These two rankings indicate that increasing chitosan to 1.0% strongly delayed disintegration, especially when combined with 1.5% HPMC. By contrast, the combination of 0.5% chitosan and 1.5% HPMC produced the most favorable high-swelling/short-disintegration behavior, particularly in F2 and F7. The swelling–disintegration relationship is visualized in Figure 2.

### 3.4. Mechanical Properties

The mechanical characterization results are summarized in Table 4.

The mechanical properties of oromucosal films are essential for successful manufacturing, transport, handling, and patient acceptability. An optimal elasticity is required: films with insufficient elasticity are more prone to cracking, while excessively elastic films may undergo undesired stretching during cutting and processing. Beyond their practical relevance, mechanical properties also provide insight into the organization of the polymeric network and can affect both film disintegration and the release of active compounds. These properties are influenced by multiple formulation and processing variables, including the type and concentration of the polymer, plasticizer, and active substance, residual solvent content, film thickness, preparation method, storage conditions (temperature and humidity), and primary packaging [9].

Elongation force is a parameter that describes both the mechanical resistance of the films and their capacity for elastic deformation. The elongation force of the films was markedly affected by the formulation composition, with values ranging from 11.84 ± 0.16 N to 23.03 ± 0.15 N (Table 4). In general, films containing higher amounts of the film-forming polymers exhibited greater resistance to deformation, indicating a stronger and more cohesive polymeric matrix. The highest elongation force was recorded for F12, followed by F4 and F11, all of which contained 1% chitosan and/or 1.5% HPMC K-4M, confirming the reinforcing effect of increased polymer concentration.

Among starch-free formulations, elongation force increased progressively with the polymer content, from 14.94 ± 0.21 N for F1 to 22.30 ± 0.26 N for F4. Increasing the HPMC concentration from 1.0% to 1.5% at constant chitosan content increased the elongation force both at 0.5% chitosan (F1 vs. F2) and at 1% chitosan (F3 vs. F4). A similar trend was observed for chitosan: at a constant HPMC concentration, films containing 1% chitosan showed higher elongation forces than those containing 0.5% chitosan (F1 vs. F3 and F2 vs. F4). These findings indicate that both chitosan and HPMC contributed positively to mechanical resistance during elongation.

In films containing 0.5% chitosan and 1% HPMC, starch addition reduced the elongation force, with the lowest value observed for F5 (11.84 ± 0.16 N). A slight increase was observed at the higher starch level in F6, although the value remained below that of the starch-free counterpart F1. F5 and F6 also exhibited the highest loss-on-drying values. Because loss on drying is not water-specific, a direct causal relationship with residual water cannot be established from the present data. A greater volatile/moisture-associated fraction may have contributed to matrix plasticization and reduced intermolecular cohesion, but this interpretation remains a hypothesis requiring water-specific measurements and controlled-humidity studies.

A similar decrease was found for formulations containing 0.5% chitosan and 1.5% HPMC, where F7 and F8 showed lower elongation forces than F2. In contrast, in films containing 1% chitosan, starch had a limited or even favorable effect. For the formulations with 1% chitosan and 1% HPMC, the elongation force remained comparable or slightly increased after starch addition (F3, F9, F10), while in the formulations containing 1% chitosan and 1.5% HPMC, the elongation force remained high and reached its maximum in F12. The literature indicates that the amylose content of starch may contribute to increased elasticity of polymeric films [29].

The results are in agreement with previous reports on starch/chitosan-based films, which showed that the tensile strength generally increases with the polymer content, particularly with an increasing chitosan concentration, due to the formation of a stronger and more cohesive polymeric network. Studies showed that the increase in starch and chitosan content led to thicker and mechanically stronger films, while the reinforcing effect of chitosan was attributed to intermolecular hydrogen bonding between starch and chitosan. However, studies showed that excessively high chitosan levels may impair mechanical performance, suggesting that the final behavior depends on the balance between intramolecular and intermolecular hydrogen bonds [31].

Thickness reduction after elongation reflects the extent of film deformation under mechanical stress and can be regarded as an indirect indicator of film ductility and structural rearrangement during stretching. In the present study, this parameter showed considerable variation among formulations, with values ranging from 15.01 ± 1.32% to 37.40 ± 2.41%. The highest thickness reduction was observed for F7, followed by F8 and F2, whereas the lowest values were recorded for F12, F10, and F11. These differences indicate marked formulation-dependent changes in the ability of the films to undergo deformation before failure.

Among the starch-free formulations, thickness reduction decreased with an increasing chitosan content, from 23.21% in F1 to 22.86% in F3 and from 34.35% in F2 to 20.27% in F4. This suggests that increasing the chitosan concentration limited post-deformation thinning, most likely by promoting a more cohesive and mechanically resistant polymer matrix. The effect of HPMC was more complex. At a low chitosan content (0.5%), increasing HPMC from 1.0% to 1.5% markedly increased the thickness reduction (F1 vs. F2), whereas at 1% chitosan, it slightly decreased this parameter (F3 vs. F4). Therefore, the influence of HPMC on film thinning appears to depend on its interaction with chitosan within the matrix.

The effect of starch was strongly dependent on the polymer composition. In films containing 0.5% chitosan, starch addition generally increased thickness reduction after elongation, particularly in F7 and F8, indicating a greater tendency toward thinning and deformation under stress. Formulations F2, F7, and F8 exhibited the highest elasticity, as evidenced by a thickness decrease exceeding 30%. This behavior appears to be favored by the higher relative proportion of HPMC compared with chitosan (chitosan:HPMC ratio of 1:3). By contrast, in formulations containing 1% chitosan, starch addition reduced the thickness reduction compared with the corresponding starch-free films, as shown by F9–F12 relative to F3 and F4. This behavior suggests that starch may act differently depending on the matrix strength: in weaker systems, it may promote structural discontinuities and facilitate deformation, whereas in stronger polymer networks, it may restrict thinning, possibly by altering chain mobility and deformation pathways.

Overall, thickness reduction after elongation proved to be a sensitive parameter for distinguishing between more deformable and more resistant films. Higher values were associated with greater susceptibility to thinning under stress, whereas lower values indicated better dimensional stability during elongation. The lowest thickness reduction observed for F12, together with its high elongation force, supports the conclusion that this formulation had one of the most mechanically resistant structures among the tested films.

Rupture resistance was strongly dependent on the formulation composition, ranging from 2.83 N/mm^2^ for F5 to 9.93 N/mm^2^ for F4. The highest value was recorded for F4, followed by F11 and F12, whereas the lowest values were observed for F5, F6, and F10. Overall, these results indicate that the resistance of the films to localized rupture was governed by the relative proportions of chitosan, HPMC, and starch.

Among the starch-free formulations, rupture resistance increased progressively with the polymer content. At 0.5% chitosan, increasing HPMC from 1.0% to 1.5% increased the rupture resistance from 7.61 (F1) to 8.46 (F2). A similar effect was observed at 1% chitosan, where rupture resistance rose from 8.84 (F3) to 9.93 (F4). Likewise, increasing the chitosan concentration at a constant HPMC content improved rupture resistance, as shown by F1 vs. F3 and F2 vs. F4. This suggests that both chitosan and HPMC contributed positively to matrix cohesion and puncture resistance. This interpretation is consistent with published work showing that HPMC-based films reinforced with chitosan display improved mechanical performance and that chitosan/HPMC blends can form structurally compatible, cohesive matrices [32].

A mechanistic explanation is also plausible. An increased chitosan content may strengthen the matrix through enhanced intermolecular interactions, while HPMC contributes to film-forming capacity and cohesion. In polymer-blend systems, such strengthening is often attributed to a denser and better-connected network structure. Chitosan-containing films have repeatedly been reported to show improved mechanical resistance, and reviews of starch–chitosan systems likewise emphasize that the final strength depends strongly on the composition and intermolecular compatibility [33].

The addition of starch generally reduced rupture resistance, but the magnitude of this effect was clearly formulation-dependent. This effect was most pronounced in the formulations with lower HPMC contents. In the F1-derived systems, rupture resistance decreased from 7.61 in F1 to 2.83 in F5 and 3.29 in F6, corresponding to reductions of 62.81% and 56.77%, respectively. A smaller decrease was observed in the F3-derived systems, where rupture resistance fell from 8.84 in F2 to 7.21 in F9 and 5.23 in F10, i.e., by 18.44% and 40.84%. By contrast, in formulations containing 1.5% HPMC, the effect of starch was pronounced: F2 decreased from 8.46 to 7.75 (F7) and 6.82 (F8), while F4 decreased only modestly to 9.46 (F11) and 9.25 (F12), corresponding to reductions of 4.73% and 6.85% relative to F4.

These trends suggest that starch weakened the films most strongly when introduced into less cohesive polymer systems, especially those with 0.5% chitosan and 1.0% HPMC. The presence of starch in F7 may have modified the organization of the chitosan/HPMC matrix by altering intermolecular interactions, polymer-chain packing, and the spatial distribution of hydrophilic groups. These changes may produce local differences in water affinity, swelling, and matrix softening, thereby promoting earlier structural destabilization compared with the starch-free F2 formulation. In contrast, polymer frameworks such as F11 and F12, which combined 1% chitosan with 1.5% HPMC, might have a denser and more cohesive polymer network with stronger intermolecular interactions. Such a matrix can distribute the applied load more effectively and better preserve structural continuity, thereby reducing the extent to which starch incorporation decreases puncture resistance. This composition-dependent behavior is consistent with broader literature on starch–chitosan films, where starch may either reinforce or weaken the system depending on polymer ratio, phase distribution, and compatibility [33].

Taken together, the puncture-strength data show that rupture resistance was primarily governed by the total polymer content and matrix organization. Formulations rich in both chitosan and HPMC exhibited the greatest resistance to puncture, whereas starch tended to reduce this parameter, particularly in structurally weaker systems.

Reported tensile strength values for commercially available oromucosal films range from 0.08 N/mm^2^ for PediaLax^®^ films to 0.36 N/mm^2^ for Risperidon Hexal^®^ films [9]. In addition, other authors have suggested minimum tensile strength values of 0.3 N/mm^2^ for oral films [19]. However, these literature values were obtained using uniaxial tensile-testing configurations and are not directly comparable with the apparent puncture-stress values determined in the present study. The current measurements were obtained by centrally loading circular films fixed around their perimeter, which generated radial and biaxial deformation before puncture. Thus, the puncture-based method provides information on multidirectional deformation, resistance to localized mechanical damage, and overall film integrity under the specified loading configuration. These characteristics may be relevant to practical film handling, but the reported values should not be interpreted as conventional tensile strength or elongation at break.

Folding endurance represents the ability of the film to withstand repeated bending at the same point without cracking or breaking and is commonly used as an indicator of film flexibility, mechanical integrity, and handling resistance. In practical terms, a high folding endurance suggests that the film is sufficiently elastic and cohesive to tolerate repeated mechanical stress during handling, packaging, and administration [34,35]. Higher folding endurance indicates greater film flexibility, which may improve the ability of the film to adhere to the buccal mucosa [36].

In the present study, folding endurance values were high for almost all formulations (Table 4). Most films withstood more than 300 folding cycles without visible cracking, indicating excellent flexibility and good structural integrity. Only F1, F5, and F6 showed values below this limit, with 285 ± 2.32, 225 ± 2.51, and 218 ± 3.43 folds, respectively. These results suggest that the majority of the formulations possessed adequate resistance to repeated bending. The lowest folding endurance values were recorded for films containing the lowest chitosan concentration (0.5%) and the lowest HPMC concentration (1.0%), particularly after starch addition. Compared with the starch-free formulation F1, the incorporation of starch in F5 and F6 further reduced the folding endurance, indicating that in this relatively weak polymer matrix, starch negatively affected film flexibility and resistance to repeated deformation. This behavior is consistent with the puncture strength data, where F5 and F6 also exhibited the lowest resistance to rupture. By contrast, increasing the concentration of HPMC and/or chitosan markedly improved the folding performance. Formulations F2, F3, and F4, which contained higher levels of one or both film-forming polymers, all withstood more than 300 folds. The same trend was maintained for the starch-containing formulations F7–F12, suggesting that when the polymer framework was sufficiently strong, the films were able to accommodate starch incorporation without losing flexibility. Thus, the effect of starch on folding endurance was strongly dependent on the initial cohesion of the polymer matrix. The results support the conclusion that formulations with higher chitosan and HPMC contents displayed superior mechanical reliability during repeated deformation. These findings are supported by literature data showing that lower contents of film-forming polymers may lead to reduced folding endurance [37].

In addition, previous studies have reported that chitosan can enhance film flexibility and mechanical integrity, suggesting that its presence may compensate, at least in part, for the higher brittleness associated with HPMC-rich systems [38].

In a study, HPMC K-4M was shown to act as the hydrophilic polymer and to enhance film flexibility, as reflected by the increase in folding endurance at higher concentrations. In contrast, ethyl cellulose, as a hydrophobic polymer, decreased the folding endurance, most likely by increasing matrix rigidity and reducing chain mobility [36].

Moreover, the glycerin used in films formulation plays a key role as a plasticizer by improving film flexibility and reducing brittleness, which may increase folding endurance. Its effect is generally attributed to the reduction of intermolecular forces between polymer chains, resulting in increased chain mobility and easier deformation under repeated bending [39].

### 3.5. Surface Roughness and Heterogeneous Hydration

**Figure 3:**
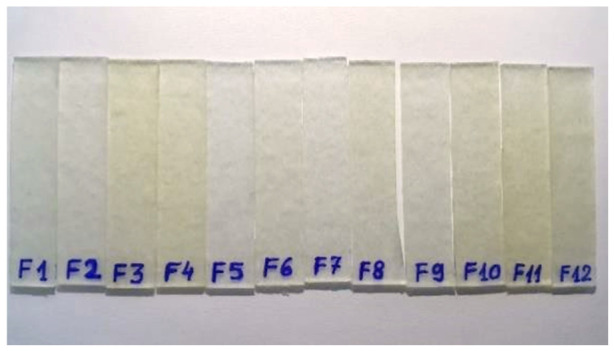
Oromucosal film samples fixed on glass support for profilometric (roughness) analysis.

**Figure 4:**
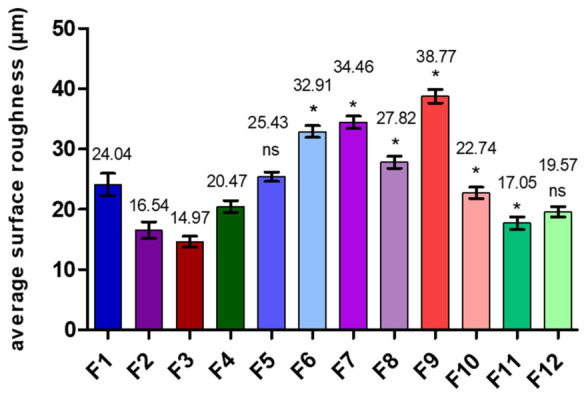
Average surface roughness (Ra) of polysaccharide-based oromucosal films (F1–F12). Data are expressed as the mean ± SD. * *p* < 0.05 versus the corresponding starch-free formulation; *ns*, not significant.

Samples prepared for profilometry are shown in Figure 3 (mounting on glass support). The mean roughness (Ra) values for all formulations are summarized graphically in Figure 4.

Surface roughness was markedly influenced by the film composition, with mean roughness (Ra) values ranging from 14.97 µm to 38.77 µm. The lowest roughness was recorded for F3, followed by F2 and F11, whereas the highest values were observed for F9, F7, and F6. These results indicate substantial formulation-dependent differences in surface topography.

Among the starch-free films, roughness remained relatively low (14.97–24.04 µm). Increasing HPMC from 1.0% to 1.5% had a different effect depending on the chitosan concentration: at 0.5% chitosan, roughness decreased (F1 vs. F2), whereas at 1% chitosan, it increased slightly (F3 vs. F4). This suggests that the surface texture was influenced by their relative ratio within the matrix.

The addition of starch generally increased surface roughness, although the extent of this effect depended strongly on the polymer background. In formulations containing 0.5% chitosan, roughness increased substantially after starch incorporation, particularly in F6 and F7. A similar trend was observed for the 1% chitosan/1% HPMC system, where F9 showed the highest roughness of all formulations (38.77 µm). By contrast, in the 1% chitosan/1.5% HPMC system, starch had only a limited effect, and F11 and F12 remained among the smoothest starch-containing films. These findings suggest that starch promoted surface heterogeneity more strongly in weaker or less cohesive polymer matrices, whereas in formulations with higher overall polymer content, especially those rich in both chitosan and HPMC, the matrix was better able to accommodate starch without a major disruption of surface uniformity.

A comparison with wetting behavior also suggests a functional relationship between roughness and hydration. Films with higher roughness, such as F6, F7, and F9, generally showed shorter wetting times than smoother films, supporting the view that a more irregular surface may facilitate initial liquid penetration by increasing the effective contact area with the aqueous medium. However, this relationship was not strictly linear for all formulations, indicating that wetting behavior was also influenced by the internal matrix organization and not only by the surface texture.

Overall, the roughness data indicate that film surface morphology was strongly dependent on the combined effect of chitosan, HPMC, and starch. Lower roughness values were associated with smoother and more homogeneous films, whereas higher values reflected a more irregular and heterogeneous surface structure, likely resulting from changes in the polymer compatibility, phase distribution, and matrix organization. Similar composition-dependent changes in surface structure have been reported for chitosan-based and starch-containing films in the literature [40].

In particular, the generally higher roughness observed for several starch-containing formulations agrees with reports that starch can increase structural heterogeneity and promote a less uniform surface organization in polymeric films. Reviews on starch/chitosan blends and starch-based films describe morphology and surface properties as strongly affected by the starch content, compatibility, and phase distribution within the matrix [41].

At the same time, smoother surfaces in some starch-free or polymer-richer formulations are also in line with previous chitosan-based film studies. For example, buccal films prepared with medium- and high-molecular-weight chitosan were described as homogeneous and flexible, suggesting that when an adequate chitosan-based network is formed, the resulting surface can remain relatively uniform. Likewise, investigations of the chitosan film surface properties have shown that the composition significantly influences topography and structural organization [42].

Studies on chitosan/HPMC-containing films have shown that combining chitosan with a second polymer modifies film structure and performance [43]. For an oromucosal film based on HPMC and chitosan, surface roughness should not be considered an isolated parameter, because mucoadhesion depends on a combination of the surface topography, wetting, swelling behavior, polymer chain mobility, and specific polymer–mucin interactions. In the dry state, a slightly to moderately rough surface may improve the initial contact with the mucosa by increasing the effective contact area and facilitating mechanical interlocking. However, if the surface is excessively rough, the contact can become non-uniform, with air gaps and poor adaptation to the mucosal surface, and the real area of intimate contact may become incomplete, which may reduce the initial adhesion. Under wet conditions, which are more relevant for the actual performance of an oromucosal film, the situation is more complex. After hydration, HPMC swells and forms a gel-like hydrated layer, which promotes intimate contact with the mucus and allows polymer chains to interpenetrate the mucin network [44]. If hydration is too extensive, excessive swelling or surface erosion may occur, weakening the film structure and reducing adhesion despite the increased roughness [45].

At the same time, chitosan plays a key role through its cationic amino groups, which can become protonated and interact electrostatically with the negatively charged residues of mucin, especially sialic acid and sulfate groups [46]. In addition to these electrostatic attractions, both HPMC and chitosan can form hydrogen bonds with mucin glycoproteins, further strengthening the mucoadhesive interface [47].

Rugosimetric analysis supports the use of a heterogeneity potential $V_{i}\left(x\right)$ rather than a homogeneous diffusion-only model. Rougher films are expected to show locally accelerated water penetration, uneven swelling-front propagation, and the earlier initiation of cracks or disintegration sites.

### 3.6. Model-Derived Parameter Proxies

**Figure 5:**
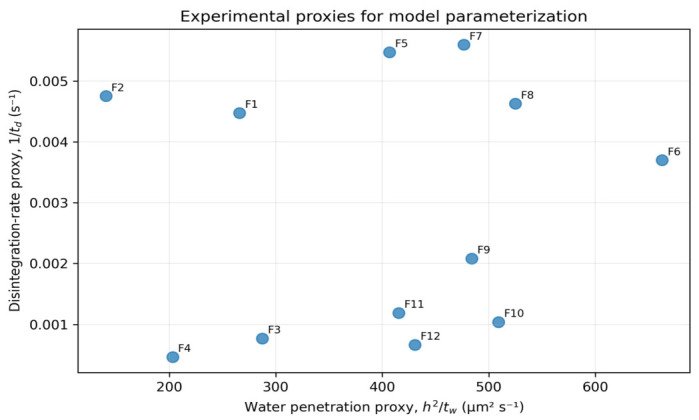
Experimental proxies for model parameterization. The horizontal coordinate is $D_{w,i}^{proxy}=h_{i}^{2}/t_{w,i}$, and the vertical coordinate is $k_{d,i}^{proxy}=1/t_{d,i}$.

**Figure 6:**
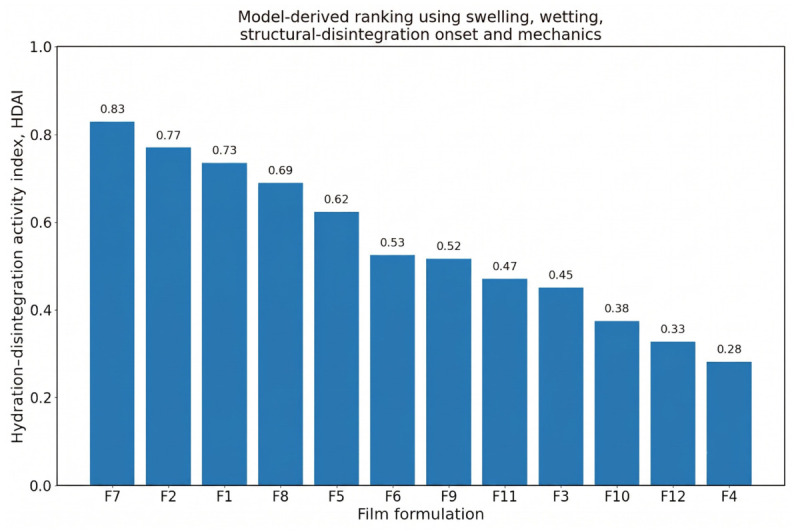
Model-derived hydration–disintegration activity index (HDAI). The index combines normalized maximum swelling and mechanical suitability with lower-is-better complementary min–max scores for the wetting time and structural-disintegration onset time.

Using Equations (3), (10), (24) and (25), model-derived proxies were calculated from the experimental means. These quantities are summarized in Table 5. The diffusivity/disintegration proxy space is shown in Figure 5, and the HDAI ranking is shown in Figure 6.

Weight sensitivity was assessed by varying each HDAI weight individually by ±20% and renormalizing the complete weight vector to sum to unity. F7 remained the highest-ranked formulation in all tested perturbations. F2 remained second in all cases except when the swelling weight was reduced by 20%, where F1 marginally exceeded F2 (0.7531 vs. 0.7528). The exact HDAI scores and the F2/F1 ordering are therefore partly weight-dependent, whereas the leading position of F7 was robust within this local sensitivity range.

### 3.7. Mechanistic Interpretation of F2 and F7

F2 and F7 represent the most relevant candidate unloaded matrix platforms for future active-compound loading and experimental release evaluation. Both contain 0.5% chitosan and 1.5% HPMC, but F7 additionally contains 0.05% starch.

F2 had a thickness of 57.2±2.9 µm, wetting time of 23.3±0.9 s, maximum swelling of 177.8±1.9%, structural-disintegration onset time of 210.3±2.2 s, rupture resistance of 8.46 ± 0.07 N/mm^2^, and folding endurance above 300 folds. It should therefore be interpreted as a high-swelling, mechanically stable, no-starch matrix.

F7 had a thickness of 80.5±4.2 µm, wetting time of 13.6±0.8 s, maximum swelling of 162.7±2.5%, structural-disintegration onset time of 178.6±3.3 s, rupture resistance of 7.75 ± 0.09 N/mm^2^, post-elongation thickness reduction of 37.40±2.41%, and folding endurance above 300 folds. F7 should therefore be interpreted as a starch-assisted, faster-wetting, high-swelling matrix with strong flexibility and slightly faster disintegration than F2.

F2 was not selected on the basis of wetting time alone. Its wetting time of 23.3 ± 0.9 s was slower than that of several formulations, but its structural-disintegration onset time of 210.3 ± 2.2 s was within the faster subset of the series and was accompanied by the highest maximum swelling and favorable mechanical integrity. These observations support its selection as a balanced unloaded matrix candidate; the direction and magnitude of any effect on API release remain to be established experimentally.

In model terms, the expected parameter relationships are as follows:

(28) $$D_{w,F7}>D_{w,F2}$$

(29) $$k_{d,F7}>k_{d,F2}$$

(30) $$D_{f,F7}>D_{f,F2}$$

The mechanistic interpretation of the F2–F7 difference should be regarded as a formulation-based hypothesis. Starch in F7 may promote earlier water entry and localized structural weakening, but the present experiments do not directly demonstrate starch-rich microdomains or internal phase discontinuities. The interpretation is nevertheless consistent with the profilometry results: mean Ra was 34.46 µm for F7 and 16.54 µm for F2, indicating greater surface irregularity in F7. This supports increased surface heterogeneity but does not by itself prove the spatial origin of the heterogeneity potential or the presence of internal starch domains.

### 3.8. Practical Film Classification

The experimental and model-derived classification is summarized as follows:

F2: high-swelling, stable, no-starch platform,

F7: rapid-wetting, starch-assisted, balanced platform,

F5,F6: rapid-wetting but mechanically fragile films,

F4,F12: strong but slow-disintegrating matrices.

Based on the integrated experimental characterization and recalculated HDAI ranking, F2 and F7 emerged as the most promising candidate unloaded matrix platforms for future active-compound loading and release testing. F2 represents a high-swelling, mechanically robust starch-free matrix, whereas F7 provides a complementary starch-containing platform characterized by faster wetting, earlier structural destabilization, high deformability, and preserved handling performance.

## 4. Discussion

The present study reframes the experimental evaluation of chitosan/HPMC/starch oromucosal films as a coupled transport, swelling, and matrix-failure problem. Conventional film characterization often generates several disconnected quality attributes: mass, thickness, pH, wetting time, swelling percentage, structural-disintegration onset time, elongation force, rupture resistance, folding endurance, and roughness. Each attribute is useful, but none alone demonstrates how an incorporated API will be released. The proposed Madelung-type multifractal framework converts these experimental descriptors into a mechanistic map of hydration and release readiness. In that map, wetting time controls the onset of water penetration, swelling capacity reflects polymer-chain hydration and relaxation, structural-disintegration onset time reports the first macroscopic failure event, mechanical parameters define resistance to deformation, and roughness contributes to heterogeneity of saliva–film contact.

From the perspective of polymer-based drug delivery, this approach complements rather than replaces classical release models. Higuchi-type diffusion, Korsmeyer–Peppas power-law fitting, Peppas–Sahlin separation of diffusional and relaxational contributions, Brazel–Peppas swelling-controlled descriptions, dissolution-profile comparison metrics, and mechanistic reviews remain essential tools for interpreting release curves from pharmaceutical matrices [48,49,50,51,52,53,54,55]. However, these models usually require experimental release data after the active compound has been loaded. The present framework addresses an earlier formulation-development question: before API loading and dissolution testing, can the unloaded-film data reported in Table 1, Table 2, Table 3, Table 4 and Table 5 support preliminary screening of matrices with hydration and structural-failure profiles potentially compatible with different release behaviors? In this sense, the model is a pre-release screening tool built on measurable pharmacotechnical and mechanical endpoints, not a validated predictor of drug release.

The main practical implication is the distinction between F2 and F7 as two useful but different unloaded matrix platforms. F2 combines 0.5% chitosan and 1.5% HPMC without starch. Its high maximum swelling, structural-disintegration onset within the faster subset of the series, and good folding endurance suggest a cohesive hydrated matrix with delayed structural failure relative to the fastest-disintegrating formulations; these findings do not establish controlled erosion or a specific release profile. By contrast, F7 contains the same chitosan and HPMC levels plus 0.05% starch and combines shorter wetting and structural-disintegration onset times with favorable flexibility. These complementary profiles justify prioritization of F2 and F7 for API-loaded experiments but do not establish drug-specific therapeutic performance.

The F2–F7 comparison also demonstrates why the framework is more informative than a simple ranking by swelling or structural-disintegration onset alone. If maximum swelling were the only criterion, F2 would be preferred; if the earliest structural-disintegration onset were the only criterion, F7 would be preferred. A practical film platform must instead satisfy a coupled constraint involving hydration, deformability, and sufficient handling integrity. The framework resolves this trade-off at the level of release readiness by separating hydration capacity, structural-failure activation, mechanical integrity, and heterogeneity. F2 is therefore interpreted as a high-swelling stable matrix candidate, whereas F7 is interpreted as a faster hydration–structural-destabilization matrix candidate.

Potential matching of film platforms to drug properties should be treated as a testable hypothesis rather than a conclusion of the present unloaded-film study. F7-like matrices may be prioritized for experiments in which rapid hydration and earlier matrix opening are desired, whereas F2-like matrices may be prioritized when cohesive swelling is of interest. The net effect on release will nevertheless depend on the API solubility, ionization, molecular size, loading level, polymer–drug interactions, and the final mucoadhesive and erosion behavior. No drug-specific preference or therapeutic-release claim is therefore made from the present data alone.

The practical value of the Madelung-type term is its ability to represent heterogeneous film behavior. Oromucosal films are not perfectly homogeneous slabs: solvent casting, drying, starch dispersion, polymer-chain entanglement, glycerin distribution, and surface roughness can generate spatially heterogeneous hydration pathways. In the model, the curvature-sensitive contribution does not imply quantum-mechanical behavior; it acts as a regularized mathematical descriptor for local focusing or dispersion of activation. Mean *Ra* is used only as a formulation-level proxy for heterogeneity amplitude and is not a direct spatial mapping of $V_{i}\left(x\right)$. Localized wetting channels, roughness-driven water entry, starch-rich regions, or microstructural defects are possible mechanistic interpretations that require spatially resolved validation. This positioning is consistent with the use of fractional, fractal, or multifractal descriptions when a polymer matrix cannot be represented adequately as a uniform continuum [56,57,58].

For formulation scientists, the framework can be used as a staged screening workflow. First, prepare unloaded candidate films and measure the same endpoints reported here: thickness, mass, wetting time, maximum swelling, structural-disintegration onset time, rupture resistance, folding endurance, and roughness. Second, calculate the formulation-level descriptors reported in Table 5; Appendix A Table A1 shows how the experimental inputs and derived descriptors enter the framework. Third, choose two or three candidate platforms with distinct mechanistic profiles rather than selecting only the single best empirical formulation. Fourth, load these candidates with the target active compound and perform release testing in simulated saliva. Finally, fit classical release equations and, where justified, the proposed PDE framework to the release data. Only after calibration and independent validation can the predictive value of the PDE parameters be assessed.

The model is also compatible with Quality-by-Design thinking. The chitosan percentage, HPMC percentage, starch percentage, glycerin level, drying conditions, casting volume, and final thickness can be treated as critical material attributes or process variables. The wetting time, structural-disintegration onset time, swelling percentage, rupture resistance, folding endurance, roughness, and release rate can be treated as critical quality attributes. The model then provides a mechanistic bridge between formulation variables and quality attributes, which is consistent with the ICH emphasis on systematic pharmaceutical development, process understanding, and risk-based control strategies [59,60]. In practical terms, the model could help define a design space where films remain thin, flexible and near-neutral in pH while achieving a target release window.

A second practical implication concerns manufacturing and scale-up. During solvent casting, small changes in the polymer concentration or drying rate can alter the viscosity, final thickness, loss-on-drying status, phase distribution and surface topography. The data reported in Table 2, Table 3 and Table 4 show that increasing polymer content improves some mechanical properties but may markedly prolong disintegration. A model-based approach can identify which variables are most likely to shift a film from rapid disintegration to excessive retention. This is useful for scale-up because industrial batches require robust performance despite minor variations in drying, solvent evaporation, casting uniformity, and environmental humidity. In this context, the model-derived descriptors could be monitored as intermediate formulation fingerprints before full release testing.

The findings also suggest how to design future API-loaded experiments. F2 and F7 should be loaded with at least two model compounds with contrasting physicochemical properties: a freely soluble compound and a poorly soluble compound. The freely soluble compound would test whether wetting and disintegration dominate release. The poorly soluble compound would test whether swelling, the residence time, and polymer-drug interactions become limiting. For each API, the cumulative release fraction should be measured in simulated saliva and fitted with zero-order, first-order, Higuchi, Korsmeyer–Peppas, Peppas–Sahlin, Weibull, and the proposed field-based model. The critical test is not whether the new model gives the highest numerical fit quality, but whether its parameters have interpretable relationships with measurable film properties. A model that fits well but produces nonphysical parameters would be less useful than a slightly less accurate model whose parameters correctly reflect hydration, swelling, and disintegration mechanisms.

The model further indicates which additional experimental endpoints would most improve predictability. Complete roughness values for all formulations would allow a quantitative calibration of the heterogeneity potential; the surface-texture information available at this stage is summarized in Appendix A Table A2. Time-resolved swelling profiles would permit estimation of water-penetration and relaxation parameters rather than relying only on maximum swelling. Imaging of hydrated cross-sections could validate the assumed one-dimensional thickness coordinate and reveal whether lateral heterogeneity is important. Mucoadhesion and residence-time measurements would be needed to move from release-readiness toward true oromucosal performance. Permeation tests across buccal or sublingual membrane models would be required for systemic-delivery claims. The additional experimental records required to convert the present release-readiness classifier into a full drug-release and absorption model are summarized in Appendix A Table A3.

The current results should therefore be interpreted as a mechanistic foundation rather than a final predictive release model. Because the films were not yet loaded with an active pharmaceutical ingredient, the model cannot claim to predict drug release quantitatively. Its contribution is upstream: it identifies which film properties matter most for release readiness and provides a mathematical vocabulary for comparing formulations before API loading. This is particularly useful for natural-polysaccharide systems, where small changes in the composition can produce nonlinear changes in hydration and mechanical failure. The proposed framework makes those nonlinearities explicit and turns empirical film characterization into a structured formulation-design problem.

## 5. Limitations and Future Work

The present work has several limitations. First, the films were not loaded with an active pharmaceutical ingredient, so the proposed release-flux equations remain prospective. Second, complete formulation-level roughness values should be tabulated explicitly; the current model uses the roughness information summarized in Appendix A Table A2. Third, the HDAI is a transparent heuristic ranking descriptor rather than a validated pharmacopoeial, regulatory, or release endpoint. Fourth, the model parameters $D_{w,i}$, $D_{f,i}$, $\gamma_{i}$, $k_{m,i}$, and $K_{i}$ require independent calibration before predictive use. Fifth, the model-derived proxies and HDAI were calculated from experimental means without the propagation of replicate-level uncertainty. Replicates do not alter the PDE structure, but they affect the precision of derived quantities and ranking stability. Sixth, the HDAI weights were specified a priori and were not optimized against release data; the local sensitivity analysis supports a robust leading position for F7 but shows that the F2/F1 ordering can change when the swelling weight is reduced.

An additional limitation of the present study is that mucoadhesion was not evaluated at this preliminary stage, although both chitosan and HPMC have been widely used in mucoadhesive delivery systems because of their recognized adhesive properties. The current work was designed to screen unloaded polymeric matrices and to identify formulations with favorable hydration, swelling, structural, and mechanical characteristics before API incorporation. Since mucoadhesive performance can be influenced not only by the chitosan/HPMC ratio, but also by the incorporated active substance, hydration state, surface properties, and final formulation structure, its experimental assessment will be more relevant after selection and loading of the most promising films. Accordingly, ex vivo mucoadhesive strength, residence time, and detachment-force measurements using an appropriate mucosal substrate will be included in the next development stage for API-loaded films, together with release, permeation, and biocompatibility studies.

Storage temperature and relative humidity were not evaluated as independent variables, and no longitudinal stability study was performed. Consequently, the present results describe the films only at the time of testing. Future studies will assess API-loaded F2 and F7 films under predefined temperature and humidity conditions at multiple time points to determine possible changes in the moisture content, polymer-network structure, mechanical properties, hydration behavior, and drug-release performance.

Future work should therefore include API-loaded F2 and F7 films, dissolution/release testing in simulated saliva, active-content uniformity, mucoadhesion testing, residence-time studies, microscopic or profilometric mapping of starch-induced heterogeneity, and fitting of both empirical and PDE-based release models. FTIR, Raman spectroscopy, and DSC should also be evaluated as complementary sources of information on polymer–polymer interactions, hydrogen bonding, bound-water states, thermal transitions, and matrix organization. Such descriptors should be incorporated into the mathematical framework only after calibration and model-selection analysis to avoid unnecessary overparameterization. The most important validation step will be to test whether calibrated model parameters track experimentally measured changes in release kinetics after controlled changes in chitosan, HPMC, and starch concentrations.

The applicability domain of the current model is limited to the investigated hydrophilic chitosan/HPMC-based films. The present model should not be extrapolated directly to hydrophobic, pH-responsive, enzyme-sensitive, or otherwise stimuli-responsive polymer systems, because these may involve different water-transport, swelling, erosion, and degradation mechanisms. Likewise, direct transfer to particulate-loaded composite films, including systems containing nanoparticles, microparticles, insoluble drug crystals, or porous carriers, is not justified without additional terms for interfacial transport, tortuosity, sedimentation, percolation, and local stress concentration.

## 6. Conclusions

This manuscript proposes a Madelung-type multifractal swelling-disintegration-release-readiness model for chitosan/HPMC/starch oromucosal films and applies it to the experimental characterization reported in Table 1, Table 2, Table 3, Table 4 and Table 5 for twelve formulations. The model reformulates the film as a thin hydrated polymer slab in which water penetration, release activation, swelling-front motion, matrix integrity, and hydration-induced loading evolve as coupled fields across the film thickness.

The practical value of the model lies in translating conventional film-quality measurements into formulation-relevant descriptors. The wetting time becomes a proxy for the onset of hydration. Maximum swelling reflects water uptake and polymer-chain relaxation. The structural-disintegration onset time reports the first macroscopic matrix-failure event. Mechanical strength and folding endurance define handling robustness, while roughness contributes to local hydration heterogeneity. Loss on drying is treated as a gravimetric formulation descriptor rather than as a water-specific measurement. Together, these quantities support the comparative interpretation of hydration, structural failure, and handling behavior but do not establish actual API-release kinetics.

The model supports the experimental selection of F2 and F7 as the most promising candidate unloaded matrix platforms, but assigns them different formulation profiles. F2 is best interpreted as a high-swelling, mechanically stable starch-free matrix candidate for future API-loading studies. F7 is best interpreted as a faster-wetting, starch-assisted matrix candidate with earlier structural destabilization. These roles are hypotheses for subsequent experimental release evaluations and should not be interpreted as validated claims of sustained or rapid drug release.

For drug-delivery development, the model provides a practical decision pathway. Candidate films can first be screened in the unloaded state, ranked by model-derived hydration and disintegration descriptors, and then selected for API loading. After active loading, classical release models and the proposed field-based model can be fitted in parallel. The approach is therefore not intended to discard standard release equations, but to add mechanistic interpretability before and after release testing.

Future validation should focus on API-loaded F2 and F7 films, full release curves in simulated saliva, mucoadhesion and residence-time testing, membrane permeation studies, and complete time-resolved swelling and roughness data records. If the model parameters remain correlated with experimentally measured release profiles after API loading, the proposed framework could become a useful tool for the rational development, design-space definition, and optimization of polymeric oromucosal films.

## Figures and Tables

**Table 1:** Experimental design of the chitosan/HPMC/starch oromucosal films.

Film	Chitosan (%)	HPMC (%)	Glycerin (%)	Starch (%)
F1	0.5	1.0	1.0	-
F2	0.5	1.5	1.0	-
F3	1.0	1.0	1.0	-
F4	1.0	1.5	1.0	-
F5	0.5	1.0	1.0	0.05
F6	0.5	1.0	1.0	0.10
F7	0.5	1.5	1.0	0.05
F8	0.5	1.5	1.0	0.10
F9	1.0	1.0	1.0	0.05
F10	1.0	1.0	1.0	0.10
F11	1.0	1.5	1.0	0.05
F12	1.0	1.5	1.0	0.10

**Table 3:** Wetting, swelling, and disintegration behavior of the films.

Film	Wetting Time (s)	Maximum Swelling (%)	Structural-Disintegration Onset Time (s)
F1	10.6 ± 1.3	134.6 ± 2.4	223.3 ± 4.3
F2	23.3 ± 0.9	177.8 ± 1.9	210.3 ± 2.2
F3	17.3 ± 0.8	112.9 ± 2.5	1300.6 ± 12.1
F4	26.6 ± 1.5	124.2 ± 3.1	2160.3 ± 23.3
F5	7.3 ± 1.2 *	121.1 ± 2.7 *	182.6 ± 4.1
F6	9.3 ± 1.3	105.9 ± 2.9 *	270.3 ± 3.5 *
F7	13.6 ± 0.8 *	162.7 ± 2.5 *	178.6 ± 3.3 *
F8	16.3 ± 1.2 *	140.9 ± 1.9 *	216.0 ± 4.7
F9	14.3 ± 0.7 *	99.3 ± 2.3 *	480.3 ± 10.6 *
F10	16.3 ± 0.8	91.9 ± 2.1 *	961.6 ± 14.4 *
F11	17.6 ± 0.8 *	102.7 ± 2.2 *	840.3 ± 11.5 *
F12	20.3 ± 1.7 *	97.5 ± 1.4 *	1510.6 ± 57.6 *

Values are expressed as the mean ± SD. Starch-containing formulations were compared with their corresponding starch-free reference: F5–F6 vs. F1, F7–F8 vs. F2, F9–F10 vs. F3, and F11–F12 vs. F4. * *p* < 0.05.

**Table 4:** Mechanical properties of oromucosal films (mean ± SD).

Film	Elongation Force (N)	Thickness Reduction After Elongation (%)	Rupture Resistance (N/mm^2^)	Folding Endurance
F1	14.94 ± 0.21	23.21 ± 2.31	7.61 ± 0.06	285 ± 2.32
F2	18.81 ± 0.32	34.35 ± 2.22	8.46 ± 0.07	>300
F3	20.54 ± 0.15	22.86 ± 1.50	8.84 ± 0.09	>300
F4	22.30 ± 0.26	20.27 ± 1.30	9.93 ± 0.11	>300
F5	11.84 ± 0.16 *	28.73 ± 1.60 *	2.83 ± 0.08 *	225 ± 2.51
F6	13.07 ± 0.15 *	25.11 ± 1.51	3.29 ± 0.06 *	218 ± 3.43
F7	16.24 ± 0.12 *	37.40 ± 1.41 *	7.75 ± 0.09 *	>300
F8	17.21 ± 0.14 *	35.82 ± 2.31	6.82 ± 0.11 *	>300
F9	19.71 ± 0.13 *	17.81 ± 1.31	7.21 ± 0.13 *	>300
F10	21.07 ± 0.14	15.22 ± 1.21 *	5.23 ± 0.15 *	>300
F11	21.97 ± 0.12	15.29 ± 1.42	9.46 ± 0.18 *	>300
F12	23.03 ± 0.15 *	15.01 ± 1.32 *	9.25 ± 0.16 *	>300

Values are expressed as the mean ± SD. Starch-containing formulations were compared with their corresponding starch-free reference: F5–F6 vs. F1, F7–F8 vs. F2, F9–F10 vs. F3, and F11–F12 vs. F4. * *p* < 0.05.

**Table 5:** Model-derived proxies and formulation regimes calculated from the experimental means.

Film	$D_{w}^{proxy}$ (µm^2^/s)	$k_{d}^{proxy}$ (s^−1^)	HDAI	Assigned Regime
F7	476.5	0.00560	0.829	balanced platform
F2	140.4	0.00476	0.770	balanced platform
F1	266.0	0.00448	0.735	intermediate
F8	524.9	0.00463	0.689	starch-accelerated matrix
F5	406.9	0.00548	0.624	fragile fast-wetting matrix
F6	662.6	0.00370	0.525	fragile fast-wetting matrix
F9	484.1	0.00208	0.517	intermediate
F11	415.4	0.00119	0.471	intermediate
F3	287.3	0.00077	0.451	slow disintegration
F10	509.2	0.00104	0.375	slow disintegration
F12	430.7	0.00066	0.328	slow disintegration
F4	203.1	0.00046	0.282	slow disintegration

## Data Availability

The original contributions presented in this study are included in the article. Further inquiries can be directed to the corresponding authors.

## Abbreviations

The following abbreviations are used in this manuscript:

ANOVA	One-way analysis of variance
API	Active pharmaceutical ingredient
HDAI	Hydration–disintegration activity index
ICH	International Council for Harmonisation
HPMC	Hydroxypropyl methylcellulose
PDE	Partial-differential-equation
SD	Standard deviation
RSD	Relative standard deviation

## Appendix A

The complete formulation, pharmacotechnical, and mechanical data are reported in the main manuscript. Specifically, the formulation matrix is reported in Table 1; loss on drying, surface pH, film mass, and film thickness are reported in Table 2; wetting, maximum swelling, and structural-disintegration onset behavior are reported in Table 3; mechanical properties are reported in Table 4; and model-derived descriptors are reported in Table 5. Appendix A therefore does not repeat these tables. Instead, Appendix A Table A1 provides a table-placement and model-use map, Appendix A Table A2 summarizes the available non-duplicated surface-texture information, and Appendix A Table A3 lists the additional experimental records required for full validation of the proposed drug-release model.

Appendix A Table A1 summarizes where each data category is reported and how it enters the proposed model. This table is intended as a traceability map rather than a repetition of the numerical values already presented in Table 1, Table 2, Table 3, Table 4 and Table 5.

**Table A1:** Table traceability map for experimental inputs and model-derived descriptors.

Data Category/Descriptor	Location in Manuscript	Primary Variables	Use in the Model
Formulation design	Table 1	Chitosan, HPMC K-4M, glycerin, starch	Defines composition-dependent heterogeneity potential and formulation classes
Basic film properties	Table 2	Loss on drying, surface pH, mass, thickness	Defines hydration readiness, geometry, and formulation feasibility
Hydration and disintegration properties	Table 3	Wetting time, maximum swelling, structural-disintegration onset time	Defines water-penetration, swelling-source, and disintegration-rate proxies
Mechanical properties	Table 4	Elongation force, post-elongation thinning, rupture resistance, folding endurance	Defines matrix-integrity, deformability, and brittleness penalties
Model-derived descriptors	Table 5	Water-penetration proxy, disintegration-rate proxy, HDAI, assigned regime	Supports mechanistic ranking and formulation classification
Surface texture/roughness	Appendix A Table A2	Available roughness ranges and qualitative trends	Supports qualitative interpretation of hydration-front heterogeneity

Appendix A Table A2 reports only the surface-texture information that was not already tabulated in the main manuscript. The available roughness information includes reported ranges and comparative effects of starch addition, but complete formulation-level numerical roughness means for all twelve films were not tabulated. For this reason, roughness was used qualitatively in the present model rather than as a fitted quantitative term.

**Table A2:** Non-duplicated surface-texture and roughness information available for model interpretation.

Film Group	Starch Content (%)	Reported Mean Roughness Information (µm)	Use in Manuscript Model
Films without starch	0	Reported range: 14.97–24.04	Qualitative baseline for surface heterogeneity and hydration-front regularity
Films with starch	0.05–0.10	Reported range: 22.74–38.77, generally higher than corresponding starch-free films, except F11 and F12	Qualitative evidence that starch increases surface heterogeneity and may accelerate localized wetting/disintegration
Individual F1–F12 roughness profiles	Formulation-dependent	Profiles available graphically; complete numerical means were not tabulated for all formulations	Recommended for future quantitative coupling to the heterogeneity potential

Appendix A Table A3 lists the additional experimental records that should be generated before the current framework is used as a fully predictive model for API-loaded oromucosal films. These records are not required for the unloaded-film classification presented here, but they are necessary for calibration of drug-specific release, mucoadhesion, and permeation terms.

**Table A3:** Recommended additional experimental records for full drug-release-model validation.

Data Record to Be Added	Recommended Measurement	Model Term Constrained	Practical Value
API-loaded release curves	Cumulative release versus time in simulated saliva or biorelevant medium	Release flux and erosion-coupled source terms	Calibrates the model for a specific active ingredient
Time-resolved swelling profiles	Swelling percentage at multiple early and late time points	Water-penetration and swelling-source functions	Separates rapid wetting from sustained matrix hydration
Mass-loss/erosion kinetics	Residual dry mass after defined hydration intervals	Matrix-integrity and erosion terms	Distinguishes swelling-controlled from erosion/disintegration-controlled release
Mucoadhesion and residence time	Detachment force, residence time under simulated salivary flow	Boundary and retention terms	Links in vitro release potential to sublingual residence
Complete profilometry data	Numerical roughness parameters for each film before and after hydration	Heterogeneity potential	Quantifies how surface texture controls local hydration and cracking
Content uniformity	API assay across film areas and batches	Initial drug distribution	Supports scale-up and quality-by-design implementation
